# Progress in Ophthalmology

**Published:** 1895-02-02

**Authors:** 


### PROGRESS IN OPHTHALMOLOGY.

### OPHTHALMOLOGY.

**Detachment of Retina.**—Bull[1] gives an interesting summary of conclusions based upon thirty-eight cases of this affection. All the patients were placed on their backs in bed, atropine was applied to one or both eyes according to the necessities of the case, a bandage was applied to the affected eye, and this treatment was maintained for from three to eight weeks. Pilocarpine was injected hypodermically in daily doses in twenty-five cases, beginning with a minute dose and increasing it to toleration. In a number of cases the drug produced such unpleasant or alarming symptoms that it was necessary to discontinue it, and experience showed that it ought not to be used for patients suffering from cardiac disease. Where it was contra-indicated, or was not borne, small doses of sodium bicarbonate and potassium iodide, largely diluted, were given in order to induce free action of the kidneys and bowels. Perchloride of mercury in small doses was given when there was iritis or choroiditis, and in all cases of extensive opacities in the vitreous. Puncture of the eyeball through the sclera, into the sub-retinal space, was done in nineteen cases, in every instance sub-conjunctivally. Division of membranous bands in the vitreous and of the detached retina, thus letting the sub-retinal fluid into the vitreous chamber, was done in seven cases. The general results were that temporary improvement, varying in duration from a few weeks to several years, was obtained in twenty-three cases. In eleven cases there was no improvement, and in four there was apparent permanent cure, with disappearance of the detachment and restoration of useful vision. The author formulates the following conclusions: (1) That no better methods of dealing with detached retina have been discovered than those which have been in use for many years, namely, rest on the back in bed, atropine, a bandage, and the internal administration of some drug which may induce absorption of the sub-retinal fluid. (2) That the continued use of pilocarpine, even in cases in which it is apparently well borne, may produce great prostration. The desired effect may sometimes be produced by small doses of bicarbonate of soda and iodide of potassium, largely diluted with water. (3) In all recent cases, puncture of the sclera subconjunctivally may do temporary good by letting out the subretinal fluid and allowing the retina to collapse, thus producing some improvement in the vision; but this improvement is generally transient, and, when membranous bands exist in the vitreous, no benefit can be expected from simple puncture. (4) Division of fixed membranous opacities in the vitreous causes little reaction, and may do positive good, even without division of the detached retina, as it reduces the danger of extension of the detachment. It is contra-indicated in cases where the vitreous opacity is vascularised, as it would certainly induce free hæmorrhage. It should never be done in an irritated or inflamed eye. (5) Division of the detached retina, which allows the sub-retinal fluid to escape into the vitreous chamber, may always be done in a quiet eye, and causes little or no reaction. If membranous bands are present in the vitreous, these should also be divided at the same time. (6) In most cases, all these operative procedures produce but temporary improvement; and in many cases no effect whatever is gained by them. (7) That Schöler's operation (injecting solution of iodine into the vitreous) ought never to be practised.

**Removal of Foreign Bodies from the Eye by a Magnet.**—Knapp[2] relates two cases, one an "ideal success," the other a failure, and founds upon them the following recommendations with regard to the conduct of the operation: (1) Make the incision obliquely through the sclerotic, because such a wound, after the operation, is firmly closed by the vitreous pressing the sharp inner lip of the inner side of the wound against the outer lip, thus favouring rapid union, preventing presentation and escape of vitreous, with their consequences, of which the later development of fanlike cords in the vitreous, starting from the scar, are particularly prejudicial to the preservation of sight. (2) During the introduction and withdrawal of the magnet keep the wound open with a sterilised platinum wire or some similar instrument. In this way the foreign body will not be easily stripped off from the magnet by the lips of the wound.

**Diagnosis of Paralysis of the Ocular Muscles.**—Dr. E. Landolt,[3] at the International Congress of Ophthalmology, read an interesting paper on the question of how best to determine the affected eye, and the affected muscle, in cases of paralytic diplopia. He places the patient, with head erect, in front of a lighted candle placed at a distance. He covers one eye, the better of the two, if there be any difference in the acuteness of vision, with a red glass, and first decides the nature of the diplopia. His first rule is: the affected eye is that in the direction of the image of which the diplopia increases. In homonymous diplopia the image of the left eye is to the left, and that of the right eye is to the right. If the diplopia increases in looking to the left, it is the left eye that is the seat of paralysis and conversely. In a case of crossed diplopia, the image of the left eye is to the right, and that of the right eye is to the left. If then the two images separate towards the left, it is the right eye that is affected, because its image is to the left. Having thus discovered the affected eye, he applies his second rule, which is, that the paralysed muscle is that which would have given to the eye the position and the direction of the false image. In homonymous diplopia, for example, with left eye affected, it is the external rectus which is paralysed, for this is the muscle which would have directed the left eye to the place of the false image, *i.e.*, to the left. In crossed diplopia due to muscular paralysis of the right eye it is the internal rectus which is paralysed, and for the same reason. These are simple illustrations of the value and of the application of the rules, but their importance is most evident in cases of vertical diplopia. If we have placed the red glass before the left eye, and the patient says that the red flame is below, and the yellow above, such a condition might be due either to paralysis of the superior oblique or the inferior rectus of the left eye, or to paralysis of the inferior oblique or the superior rectus of the right eye. According to Landolt, if the vertical diplopia increases in looking upwards it is the right eye that is affected because its image is the higher. If the diplopia diminishes in looking upwards the right eye is not affected, and attention must be directed to the other. Suppose that the difference in height of the two images increases on looking downwards, then, since it is the left eye whose image is the lower, it is evidently this eye that is paralysed. The second law teaches us that it is one of the depressors of this eye that is in fault, because it is the depressors that direct the eye towards the image that is downwards. If the patient says that the red flame is towards the left (homonymous diplopia), but that its upper extremity leans towards the right, the law permits us at once to recognise a paralysis of the superior oblique, because this muscle not only draws the eye downwards, but also directs it outwards, and turns it around its antero-posterior axis in such a way that the upper extremity of its vertical meridian inclines towards the nose. The converse situation and direction of the image would indicate paralysis of the inferior rectus. In the discussion which followed the paper, Dr. Savage described his method of determination as follows: Holding a lighted candle in the median plane, it may be seen as one; on moving it to the patient's right, diplopia is produced. The affected muscle is on the same side of the eye to which it belongs as is the doubled candle; therefore, in the case supposed, it must be either the internal rectus of the left or the external rectus of the right eye. The affected eye sees the candle farthest removed, and occlusion first of one and then of the other quickly settles the question. If it be the superior or inferior rectus of one eye that is affected, and if, beginning on the horizontal plane, moving the candle upwards produces diplopia, it is a superior rectus that is diseased, and it belongs to the eye that sees the higher candle.

[1] Transactions of American Ophthalmological Society, 1884. [2] Op. cit
[3] The Medical Week, Aug. 10, 1894.

